# Ternary Complexes of pDNA, Neuron-Binding Peptide, and PEGylated Polyethyleneimine for Brain Delivery with Nano-Bubbles and Ultrasound

**DOI:** 10.3390/pharmaceutics13071003

**Published:** 2021-07-01

**Authors:** Yoko Endo-Takahashi, Ryo Kurokawa, Kanako Sato, Nao Takizawa, Fumihiko Katagiri, Nobuhito Hamano, Ryo Suzuki, Kazuo Maruyama, Motoyoshi Nomizu, Norio Takagi, Yoichi Negishi

**Affiliations:** 1 Department of Drug Delivery and Molecular Biopharmaceutics, School of Pharmacy, Tokyo University of Pharmacy and Life Sciences, 1432-1 Horinouchi, Hachioji, Tokyo 192-0392, Japan; y141064@toyaku.ac.jp (R.K.); y134120@toyaku.ac.jp (K.S.); y124128@toyaku.ac.jp (N.T.); nhamano@toyaku.ac.jp (N.H.); 2 Department of Clinical Biochemistry, School of Pharmacy, Tokyo University of Pharmacy and Life Sciences, 1432-1 Horinouchi, Hachioji, Tokyo 192-0392, Japan; katagairi@toyaku.ac.jp (F.K.); nomizu@toyaku.ac.jp (M.N.); 3 Laboratory of Drug and Gene Delivery Research, Faculty of Pharma-Sciences, Teikyo University, 2-11-1 Kaga, Itabashi-ku, Tokyo 173-8605, Japan; r-suzuki@pharm.teikyo-u.ac.jp; 4 Laboratory of Theranostics, Faculty of Pharma-Sciences, Teikyo University, 2-11-1 Kaga, Itabashi-ku, Tokyo 173-8605, Japan; maruyama@pharm.teikyo-u.ac.jp; 5 Department of Applied Biochemistry, School of Pharmacy, Tokyo University of Pharmacy and Life Sciences, 1432-1 Horinouchi, Hachioji, Tokyo 192-0392, Japan; takagino@toyaku.ac.jp

**Keywords:** nanobubble, ultrasound, brain, gene delivery

## Abstract

In brain-targeted delivery, the transport of drugs or genes across the blood−brain barrier (BBB) is a major obstacle. Recent reports found that focused ultrasound (FUS) with microbubbles enables transient BBB opening and improvement of drug or gene delivery. We previously developed nano-sized bubbles (NBs), which were prepared based on polyethylene glycol (PEG)-modified liposomes containing echo-contrast gas, and showed that our NBs with FUS could also induce BBB opening. The aim of this study was to enhance the efficiency of delivery of pDNA into neuronal cells following transportation across the BBB using neuron-binding peptides. This study used the RVG-R9 peptide, which is a chimeric peptide synthesized by peptides derived from rabies virus glycoprotein and nonamer arginine residues. The RVG peptide is known to interact specifically with the nicotinic acetylcholine receptor in neuronal cells. To enhance the stability of the RVG-R9/pDNA complex in vivo, PEGylated polyethyleneimine (PEG-PEI) was also used. The ternary complexes composed of RVG-R9, PEG-PEI, and pDNA could interact with mouse neuroblastoma cells and deliver pDNA into the cells. Furthermore, for the in vivo experiments using NBs and FUS, gene expression was observed in the FUS-exposed brain hemispheres. These results suggest that this systemic gene delivery system could be useful for gene delivery across the BBB.

## 1. Introduction

Gene therapies are expected to be valuable for the treatment of intractable central nervous system (CNS) diseases, such as neurodegenerative disorders, malignant brain tumors, brain infections, and brain injury. In gene delivery to the brain, the main obstacle for realizing clinical application is the poor efficiency of delivery due to the blood–brain barrier (BBB), similar to drug delivery. However, the improvement of transfection efficiency into the target cells following transportation across the BBB is also essential. Recently, invasive and noninvasive approaches for brain-targeting delivery have been actively investigated. Direct injection methods, such as intraparenchymal, intrathecal, and intracerebroventricular injection, are effective in increasing the concentration locally in the brain; however, it has the risk of causing infection. Contrarily, as noninvasive methods, nanocarriers targeted to the receptors on brain capillary endothelial cells, such as transferrin receptor, insulin receptor, or low-density lipoprotein receptor related protein, have been well employed to deliver to CNS [1,2,3]. Aside from brain-specific nanocarriers, the direct delivery from nasal cavity to brain and the utilization of the response to a glycemic change were also reported as methods to enhance the efficiencies of delivery to the brain tissue [4,5,6,7,8].

Recently, it was reported that the use of focused ultrasound (FUS)-mediated microbubbles enabled a transient BBB opening in a localized brain region and enhanced drug or gene delivery effects only at FUS-exposed sites [9,10,11,12,13,14,15,16,17,18]. We also reported that the combination of nano-sized bubbles (NBs), developed on the basis of polyethylene glycol (PEG)-modified liposomes, and FUS could serve to increase the permeability of the BBB and the delivery effects of oligonucleotides or pDNA [19]. We previously showed that NBs were valuable for gene and nucleic acid delivery to muscles, tumors, ischemic muscles, and joint synovium [20,21,22,23,24,25,26,27,28,29]. In this study, we attempted gene delivery to the brain by the combination of NBs, FUS, and brain-targeted polyplexes. The combination of physical energy and targeting nanocarriers was expected that it could lead to efficient gene delivery to limited target sites in the brain tissue without overdose. For the preparation of polyplexes with brain-targeting ability, we used a rabies virus glycoprotein (RVG) peptide fused to nonamer arginine residues. RVG binds to the nicotinic acetylcholine receptor (nAchR) on neuronal cells [30,31]. RVG peptide containing nine arginine residues to the carboxy terminus was reported as a useful siRNA carrier to the brain across the BBB via systemic administration, and the combination of RVG peptide with various carriers for drug and gene delivery to the CNS was further reported [32,33,34,35]. However, perhaps because of the difference in molecule size, polyplexes of RVG peptide and pDNA have not been well reported without modifications for condensation or stability of polyplexes [36,37,38]. In this study, a ternary complex was prepared using PEGylated polyethyleneimine (PEG-PEI) to improve the in vivo stability of the polyplex. In addition, the combination of ternary complexes and BBB opening by NBs and FUS was expected to increase the efficiency of gene delivery to neuronal cells at FUS-exposed sites. We investigated the targeting ability of ternary complexes to neuronal cells and the effects of gene delivery to the brain tissue by ternary complexes with NBs and FUS.

## 2. Materials and Methods

### 2.1. Materials

The plasmid pcDNA3-Luc, derived from pGL3-basic (Promega, Madison, WI, USA) is an expression vector encoding the firefly luciferase gene under the control of a cytomegalovirus promoter. YOYO-1 iodide was purchased from Thermo Fisher Scientific (Waltham, MA, USA). Methoxy poly(ethylene glycol)-*b*-polyethyleneimine (PEG-PEI, 10,000:800 Da) was purchased from Akina, Inc. (West Lafayette, IN, USA). The lipids for NBs, 1,2-distearoyl-*sn*-glycero-3-phosphatidylcholine (DSPC), and N-(carbonyl-methoxypolyethyleneglycol 2000)-1,2-distearoyl-*sn*-glycero-3-phosphatidylethanolamine (DSPE-PEG), were purchased from NOF Corporation (Tokyo, Japan). A 200 nm polycarbonate membrane was purchased from Advantec Toyo Roshi Kaisha, Ltd. (Tokyo, Japan). Perfluoropropane gas was obtained from Takachiho Chemical Inc. Co. Ltd. (Tokyo, Japan).

### 2.2. Preparation of Ternary Complexes

RVG-R9, the RVG peptide conjugated to oligoarginine (YTIWMPENPRPGTPCDIFTNSRGKRASNG-G-RRRRRRRRRR) was synthesized manually using a 9-fluorenylmethoxycarbonyl (Fmoc)-based solid-phase strategy, prepared in the COOH terminal amide form, and purified by reverse-phase, high-performance liquid chromatography. The RVG peptide without oligoarginine was synthesized in the same way as the RVG-R9. The peptides were characterized using an electrospray ionization mass spectrometer (Xevo G2-XS QTof LC/MS, Waters, Milford, CT, USA) at the Central Analysis Center, Tokyo University of Pharmacy and Life Sciences. The pDNA solution was added to a mixed solution of PEG-PEI and RVG-R9 in an equal volume under vortexing and incubated for 10 min at room temperature to prepare ternary complexes. Each solution was prepared in HEPES-buffered glucose (HBG: 5% glucose, 10 mM HEPES, pH 7.0) and mixed at various N/P ratios, which is the ratio of basic amine groups of PEG-PEI and RVG-R9 to the phosphate groups of pDNA. The ternary complex formation was confirmed using 1% agarose gel electrophoresis. The gel was stained with GelRed (Biotium, Fremont, CA, USA) for visualization. Particle sizes and zeta potentials were measured using a Zetasizer Nano-ZSP (Malvern Panalytical, Malvern, UK). For the quantitative assay to show the percentage of complexed pDNA, we used Qubit dsDNA HS Assay Kit (Thermo Fisher Scientific, Waltham, MA, USA) and the fluorescence was measured at 485/530 nm.

### 2.3. Cell Cultures

Mouse neuroblastoma cell line, Neuro2a (JCRB Cell Bank, Osaka, Japan) was cultured in Eagle’s minimal essential medium with non-essential amino acids (FUJIFILM Wako Pure Chemical Corporation, Osaka, Japan) and supplemented with 10% heat-inactivated fetal bovine serum (Equitech Bio Inc., Kerrville, TX, USA), 100 U/mL penicillin, and 100 μg/mL streptomycin in a humidified atmosphere containing 5% CO_2_ at 37 °C. The human cervical carcinoma cell line, HeLa (RIKEN BRC, Tsukuba, Japan) was cultured in Dulbecco’s modified Eagle medium (FUJIFILM Wako Pure Chemical Corporation, Osaka, Japan) supplemented with 10% heat-inactivated fetal bovine serum (Equitech Bio Inc., Kerrville, TX, USA), 100 U/mL penicillin, and 100 μg/mL streptomycin in a humidified atmosphere containing 5% CO_2_ at 37 °C. HeLa cells with low expression levels of nAChR were used as negative control cells which did not bind to the RVG peptide [32].

### 2.4. Gene Transfection with Ternary Complexes In Vitro

The day before the experiment, Neuro2a and HeLa cells (5 × 10^4^ cells/well) were seeded in a 48-well plate. Cells were incubated with 100 μL of the ternary complexes (pDNA 1 μg) in 400 μL of OPTI-MEM for 4 h at 37 °C, washed, and cultured in fresh medium for 2 days. Similarly, gene transfection using Lipofectamine 2000 (LF2000, Thermo Fisher Scientific, Inc.) was also performed as a control. The cell lysate was prepared using a lysis buffer (0.1 M Tris-HCl [pH 7.8], 0.1% Triton X-100, and 2 mM EDTA). Luciferase activity was measured using a luciferase assay system (Promega) and a multimode plate reader (Synergy HTX, BioTek Japan, Tokyo, Japan). The activity was expressed as relative light units (RLU) per mg of protein. In the inhibitory assay, cells were incubated with RVG peptide without oligoarginine for 1 h prior to transfection.

### 2.5. Cellular Specific Binding of Ternary Complexes

Neuro2a and HeLa cells (2 × 10^4^ cells/well) were seeded in a 96-well black plate (Greiner Bio-One GmbH, Kremsmünster, Austria) to evaluate the cellular specific binding of the ternary complexes, one day before the experiment. The cells were treated with the ternary complexes (YOYO-1-labeled pDNA 0.4 μg) for 2 h at 37 °C. After incubation, the cells were washed and fixed with 4% paraformaldehyde for 20 min, and the nuclei were stained with DAPI. Fluorescence was observed using a fluorescence microscope (BZ-X700; Keyence, Osaka, Japan).

### 2.6. Preparation of Liposomes and NBs

Liposomes were prepared using the reverse phase evaporation method as described previously [20]. DSPC and PEG2000 were mixed at a molar ratio of 94:6, and dissolved in 1:1 (*v/v*) chloroform/diisopropylether. HBG was added to the lipid solution, sonicated, and then evaporated. The organic solvent was removed completely, and the size of the liposomes was adjusted to less than 200 nm using extrusion equipment and a sizing filter. Thereafter, the liposomes were filter-sterilized using a 0.45 μm syringe filter (AGC TECHNO GLASS Co., Shizuoka, Japan).

NBs were prepared using liposomes and perfluoropropane gas. First, 2 mL sterilized vials containing 0.8 mL of liposome suspension (total lipid concentration: 1 mg/mL) were filled with perfluoropropane gas, capped, and then pressurized with 3 mL of perfluoropropane gas. The vials were placed in a bath sonicator (40 kHz, Bransonic M2800; Branson Ultrasonics Co., Danbury, CT, USA) for 5 min to form NBs. The mean size of the NBs was determined using a light scattering method with a zeta potential/particle sizer (Nicomp 380ZLS, Particle Sizing Systems, Santa Barbara, CA, USA).

### 2.7. Extravasation of Evans Blue Dye

In the previous report, extravasation of Evans blue dye (EB) was evaluated [19]. Mice were anesthetized and the hair was removed from the head using a depilatory cream. EB was injected intravenously into ICR mice (male, 5 weeks old) (100 mg/kg). Five minutes after the injection, NBs (100 μg/mouse) were also injected intravenously, and the right hemisphere was concurrently exposed to FUS (0.05–0.4 kW/cm^2^, 30% duty (sec), 90 s) transdermally. Sonitron GTS and HIFU transducer (20 mm diameter, 2.2 MHz, 19 mm focal depth) (Nepa Gene Co., Ltd., Chiba, Japan) were used as an ultrasound transducer. The FUS transducer was set and fixed at a distance slightly shorter than the focal depth from the head of the mouse, and the space between them was filled with water. The mice were perfused with PBS, 3 h after FUS exposure. The isolated brain tissues were divided into the right and left hemispheres, soaked in formamide solution (5 μL/mg tissue), and incubated for 24 h at 55 °C. The extracted dye concentration was determined using a spectrophotometer at 620 nm, and the non-exposed left hemispheres were used as controls.

### 2.8. In Vivo Gene Delivery to the Brain Tissue

The solution of ternary complexes (pDNA 48 μg, N/P 10, PEG-PEI:RVG-9R = 8:2) and NBs (100 μg/mouse) were injected into the mice tail vein, and right hemisphere was concurrently exposed to FUS with various conditions. The setup condition of the duty cycle using Sonitron GTS was described as (sec) or (msec) to distinguish between seconds on/off and milliseconds on/off. One day after the transfection, the tissues (brain, heart, liver, kidney, lung, and spleen) were collected and tissue homogenates were prepared with a lysis buffer. The isolated brain tissues were divided into the right and left hemispheres, and the right hemisphere exposed to FUS was analyzed. The activity is indicated as RLU per gram of tissue.

### 2.9. Statistical Analyses

All data were presented as mean ± SD (*n* = 3–6). Data were considered significant at *p* < 0.05. One-way ANOVA was used to calculate the statistical significance.

## 3. Results

### 3.1. The Formation of Ternary Complexes Composed of pDNA, PEG-PEI, and RVG-R9 Peptide

**Figure 1:**
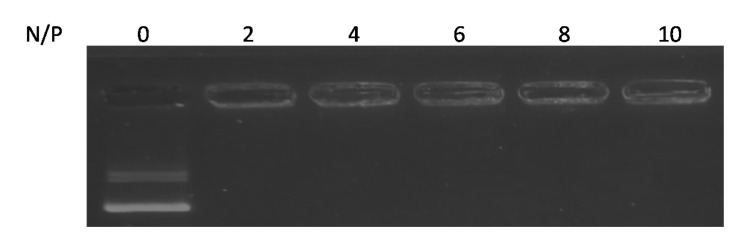
The formation of ternary complexes. A fixed amount of pDNA was incubated with various amounts of cationic polymer and peptide (PEG-PEI:RVG-R9 = 1:1). The ternary complexes were analyzed using agarose gel electrophoresis.

We initially attempted to prepare ternary complexes containing basic amine groups of PEG-PEI and RVG-R9 at a molar ratio of 1:1. As a result, the ternary complexes were almost completely formed at N/P ratios of at least 2 (Figure 1). N/P ratios > 4 have complex mean sizes of approximately 100 nm with positive zeta potentials (Table 1).

### 3.2. The Transfection Effects of Various Ternary Complexes In Vitro

**Figure 2:**
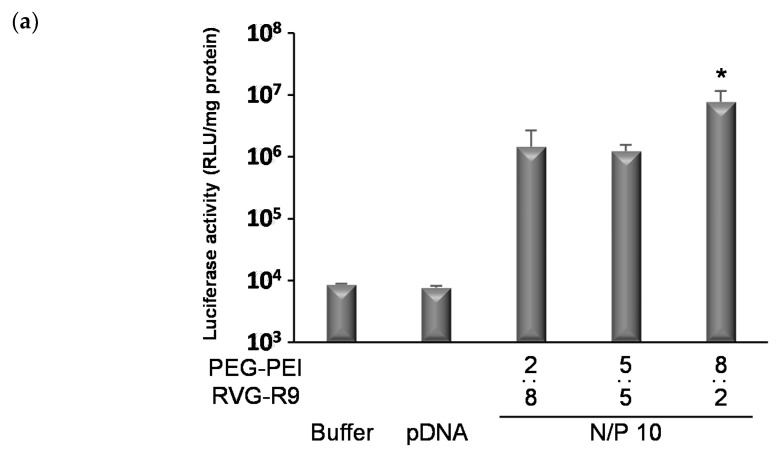

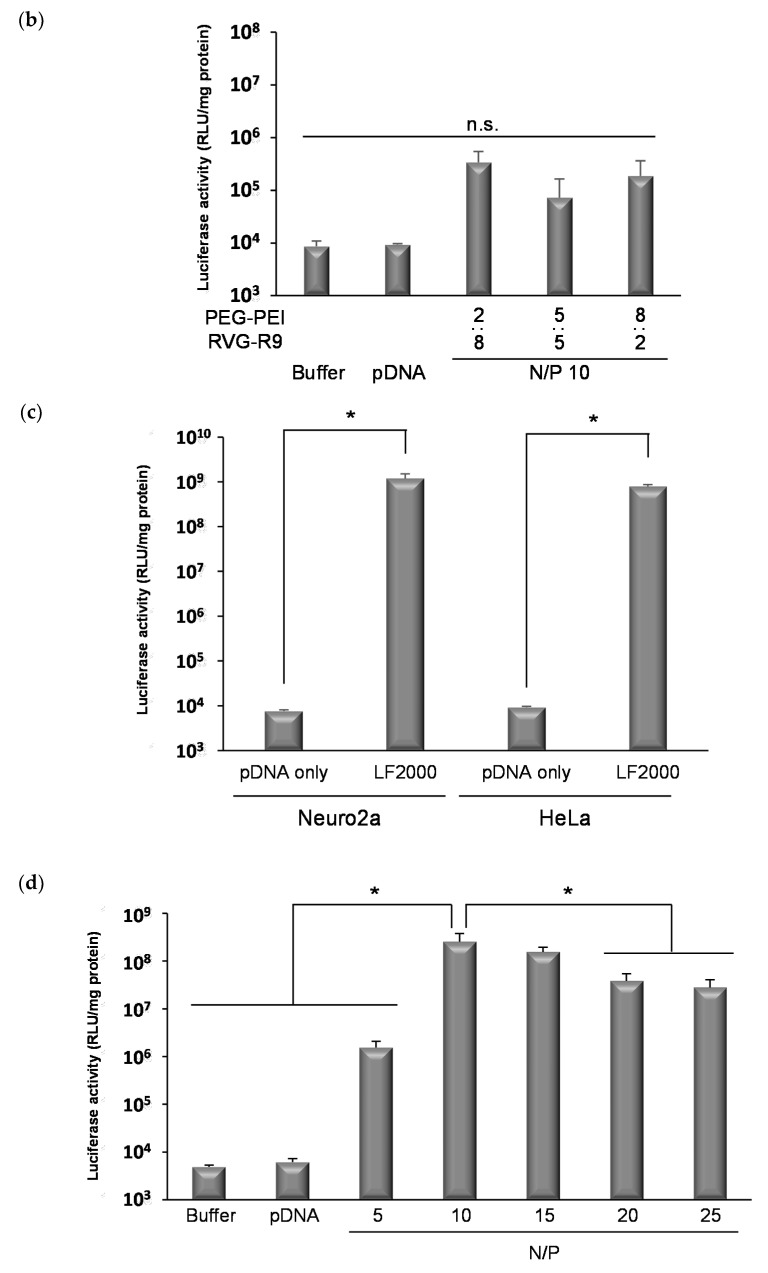
Gene transfection effects with various ternary complexes. Cells (5 × 10^4^ cells /well) were incubated with ternary complexes containing pDNA (1 μg/well) for 4 h. Luciferase activity was analyzed 2 days after the transfection. The influence of ratio of cationic polymer in ternary complexes on the transfection in (**a**) Neuro2a cells and (**b**) HeLa cells. (**c**) Gene transfection effects with LF2000. Neuro 2a cells or HeLa cells were incubated with LF2000 complexes containing pDNA (1 μg/well) for 4 h. Luciferase activity was analyzed 2 days after the transfection. The bars show the mean and S.D. (*n* = 3). * indicates *p* < 0.05 using an unpaired *t*-test. (**d**) The influence of N/P ratio of ternary complexes (PEG-PEI:RVG-R9 = 4:1) on the transfection in Neuro2a cells. The bars show the mean and S.D. (*n* = 3). * indicates *p* < 0.05 using a one-way ANOVA with Tukey’s post-hoc test. n.s. indicates no significant difference.

The N/P ratio was optimized based on the transfection effects on neuronal cells. The influence of the ratio of PEG-PEI to RVG-R9 on transfection was also confirmed. As a result, there was a significant increase in the transfection effects with the ternary complexes containing more PEG-PEI than RVG-R9 (N/P = 10, PEG-PEI:RVG-R9 = 8:2) (Figure 2a). With an increase in the PEG-PEI ratio, the complexes tended to slightly enlarge and cover the charge (Table 2, Figure S1). In HeLa cells with low expression levels of the nAch receptor, gene transfection effect by the ternary complexes was not significantly observed, although the effect by LF2000 was comparable with that in Neuro2a (Figure 2b,c). Furthermore, the transfection effects with the ternary complexes (PEG-PEI:RVG-R9 = 8:2) were increased until the N/P ratio was 10 and decreased at an N/P ratio of more than 10 (Figure 2c). At the N/P ratio of 10 (PEG-PEI:RVG-R9 = 8:2), no remarkable damage was observed, and the cell viability after transfection was approximately 80% (Figure S2). Based on these results, the ternary complexes were used at an N/P ratio of 10 (PEG-PEI:RVG-R9 = 8:2) in subsequent experiments.

### 3.3. Cellular Specificity of Ternary Complexes

**Figure 3:**
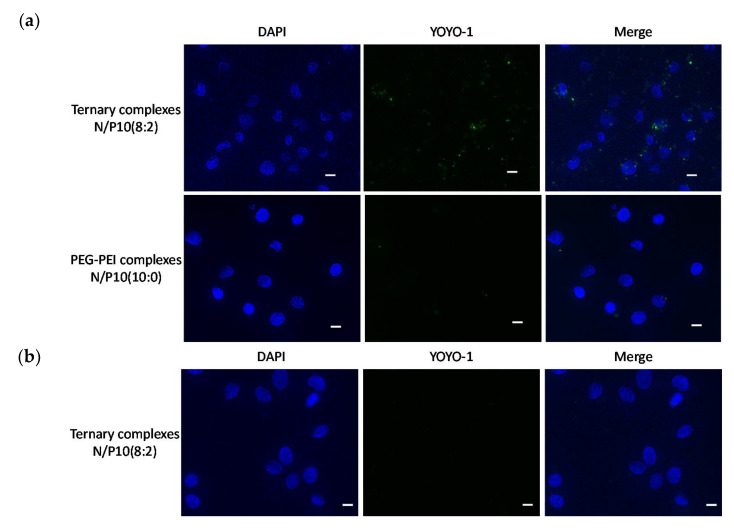

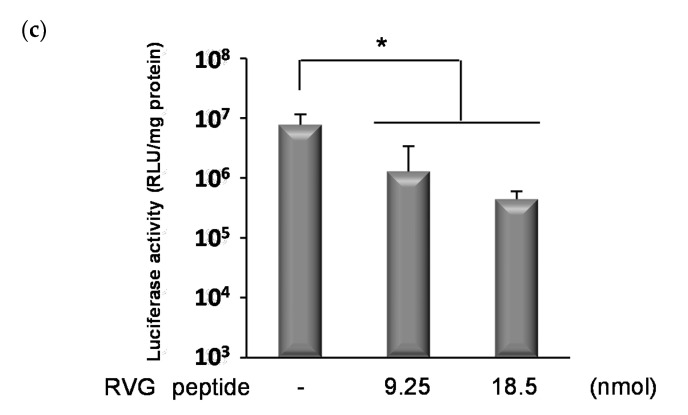
Specific interaction with ternary complexes and Neuro2a cells. (**a**) Neuro2a cells and (**b**) HeLa cells were incubated with ternary complexes containing YOYO-1 (green) labeled pDNA (0.4 μg/well) for 2 h. Cells were fixed, stained with DAPI (blue), and evaluated by fluorescence microscopy. Scale bars represent 10 μm. (**c**) The influence of preincubation with RVG peptide for 1 h on the transfection by ternary complexes (pDNA 1 μg/well, N/P 10, PEG-PEI:RVG-R9 = 8:2). The bars show the mean and S.D. (*n* = 3). * indicates *p* < 0.05 using a one-way ANOVA with Tukey’s post-hoc test.

We examined and compared the specific interaction of ternary complexes with Neuro2a cells to that of HeLa cells. As shown in Figure 3a, the interaction of ternary complexes was observed with Neuro2a cells, while less interaction of polyplexes without RVG-R9 was observed. Furthermore, the interaction of ternary complexes was also observed less with HeLa cells (Figure 3b). The inhibition assay by preincubation of the RVG peptide above the number of molecules used for the transfection showed a dose-dependent decrease in gene expression (Figure 3c). These results suggested that the ternary complexes interacted specifically with Neuro2a cells by the RVG peptide.

### 3.4. Permeability of the BBB Induced by NBs and FUS

**Figure 4:**
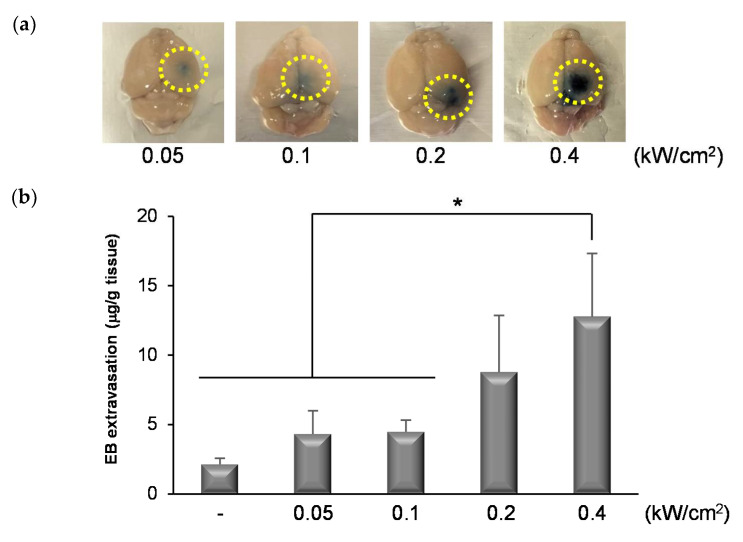
EB extravasation in the right brain (FUS-exposed site). (**a**) Distribution of FUS-induced BBB opening indicated by the extravasation of EB. (**b**) The amount of EB extravasation in the right brain. The EB was injected into the mice tail vein (100 μg/g·b.w.). After 5 min, they were injected with NBs (100 μg/mouse) and exposed to FUS with 30% duty (sec) for 90 s. After 3 h, the right brain was collected. The bars show the mean and S.D. (*n* = 3–6). * indicates *p* < 0.05 using a one-way ANOVA with Tukey’s post-hoc test.

We previously reported that the combination of NBs and FUS could enhance the permeability of the BBB and deliver nucleic acids to the focused brain site [19]. In this study, we attempted to use other equipment and apply FUS at low intensity. The mean diameter and the zeta potential of NBs were almost the same as those of NBs previously reported (619.9 ± 235.5 nm, −4.56 ± 1.83 mV). As shown in Figure 4a, the distribution of BBB disruption by EB extravasation was locally observed at the FUS-exposed site although not at the non-exposed site. The amount of EB extravasation increased in an FUS intensity-dependent manner (Figure 4b). These results suggest that BBB permeability can be enhanced by treatment of NBs with FUS.

### 3.5. In Vivo Gene Transfection by Ternary Complexes with NBs and FUS

**Figure 5:**
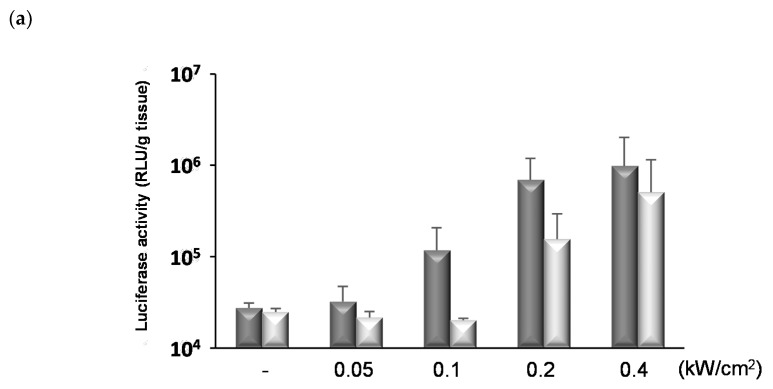

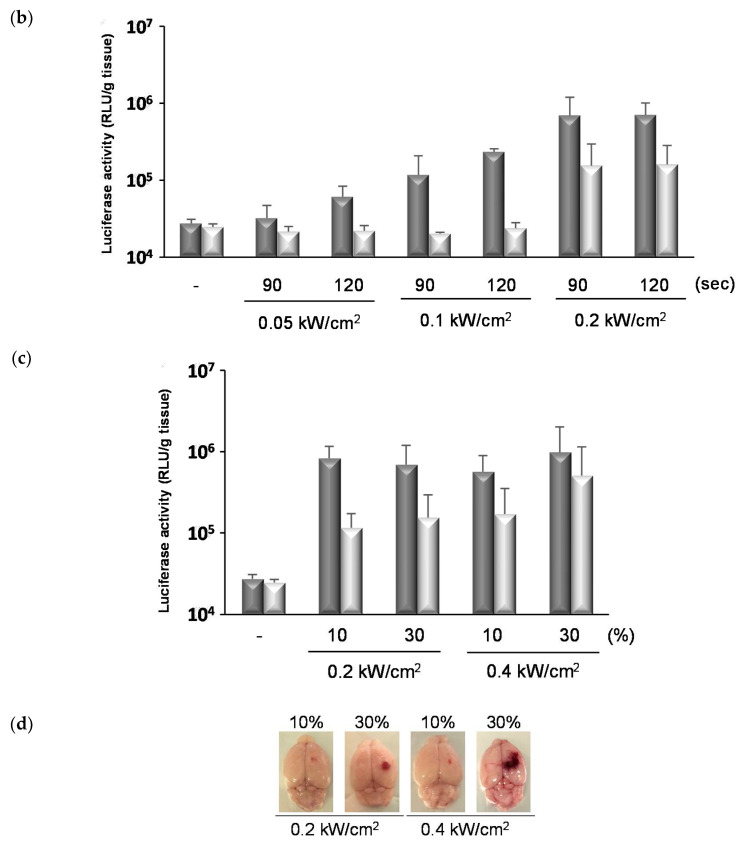
The effects of FUS conditions on the gene transfection effects into the brain tissue. The ternary complexes (pDNA 48 μg/mouse, N/P 10, PEG-PEI:RVG-R9 = 8:2) and NBs (100 μg/mouse) were injected into the tail vein of mice and the right hemisphere was concurrently exposed to FUS. The FUS conditions for each study are as follows: (**a**) intensity: 0.05–0.4 kW/cm^2^, duty: 30% (sec), time: 90 s, (**b**) intensity: 0.05–0.2 kW/cm^2^, duty: 30% (sec), time: 90 or 120 s, (**c**,**d**) intensity: 0.2 or 0.4 kW/cm^2^, duty: 10 or 30% (sec), time: 90 s. One day after the transfection, the brain tissues were collected and the luciferase activity was analyzed. The bars show the mean and S.D. (*n* = 3–6). Dark gray column: the right hemisphere exposed to FUS. Light gray column: the left hemisphere unexposed to FUS. (**d**) The tissue damages by NBs and FUS 1 day after the transfection.

**Figure 6:**
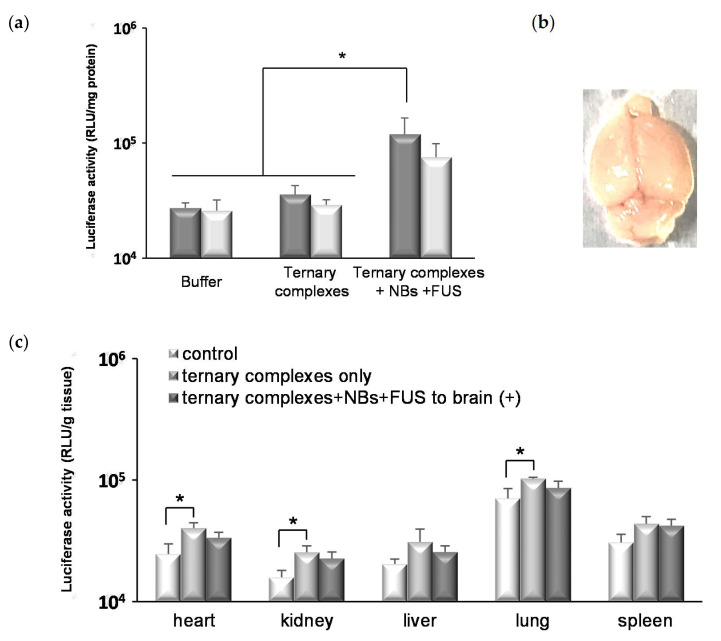
The effects of systemic gene delivery with the ternary complexes, NBs, and FUS. The ternary complexes (pDNA 48 μg/mouse, N/P 10, PEG-PEI:RVG-R9 = 8:2) and NBs (100 μg/mouse) were injected into the mice tail vein and the right hemisphere was concurrently exposed to FUS (intensity: 0.4 kW/cm^2^, duty: 10% (msec), time: 90 s). One day after the transfection, various tissues were collected and the luciferase activities of (**a**) brain, (**c**) heart, kidney, liver, lung, and spleen were analyzed. (a) Dark gray column: the right hemisphere exposed to FUS. Light gray column: the left hemisphere unexposed to FUS. The bars show the mean and S.D. (*n* = 3–4). * indicates *p* < 0.05 using a one-way ANOVA with Tukey’s post-hoc test. (**b**) The image of brain one day after the transfection by the combination of ternary complexes, NB, and FUS (intensity: 0.4 kW/cm^2^, duty: 10% (msec), time: 90 s).

We investigated the influence of FUS conditions on gene delivery. As shown in Figure 5a, the gene expression was enhanced by increasing the intensity of FUS (30% duty (sec), 90 s) compared to the unexposed control group. However, the part of mice treated with FUS at 0.2 and 0.4 kW/cm^2^ showed a hemorrhage in the brain tissues at the FUS-exposed site and the luciferase activities in the left hemispheres unexposed to FUS were enhanced on increasing the intensity of FUS. Furthermore, the influence of exposure time and duty cycle was also evaluated. As a result, the prolongation of exposure time enhanced the luciferase gene expressions; however, the gene expression levels remained low at an intensity below 0.2 kW/cm^2^ (Figure 5b). It was shown that the frequency and area of hemorrhage were moderated by the reduction of the duty cycle from 30% (sec) to 10% (sec) without a decrease in gene expression (Figure 5c,d). Thereafter, the pulse repetition time was shortened, from 1/9 s on/off to 1/9 milliseconds on/off, without changing the duty cycle at 10%. As shown in Figure 6a, the luciferase gene expression level in the group treated with the combination of ternary complexes, NBs, and FUS was significantly higher than that of the control group. Furthermore, no hemorrhage was observed in the brains of all the treated mice (Figure 6b). In the tissues not exposed to FUS, there was no difference in the luciferase expression levels between the control group treated with buffer and the treated group with ternary complexes, NBs, and FUS to the brain (Figure 6c). In the group treated with ternary complexes only, the luciferase activities of the heart, kidney, and lung were significantly enhanced compared to that of the control group, although the levels were low. However, these were slightly reduced by the exposure of FUS to the brain. These results suggest that the ternary complexes using RVG peptide and PEG-PEI could allow for less-invasive brain-targeted gene delivery by the combination of NBs and FUS.

## 4. Discussion

The improvement of the efficiency of gene delivery to neuronal cells is extremely important for the development of gene therapies for CNS diseases. Recent reports have found that microbubbles and FUS could induce a transient BBB opening [9,10]. In a clinical setting, microbubbles and state-of-the-art ultrasound devices are used to deliver chemotherapeutic agents utilizing a transient BBB opening for a patient with a malignant brain tumor. For gene therapies, delivery into targeted cells is essential in addition to transport across the BBB. In this study, we utilized the RVG-peptide and PEG-PEI to achieve this aim. Initially, the ternary complexes were optimized for gene delivery to neuronal cells. Stable complexes, which are around 100 nm, could be formed at N/P ratios of 4 or larger. As a result of the agarose gel electrophoresis, we considered that the ternary complexes were almost completely formed at N/P ratios of at least 2 by the absence of band of free pDNA at N/P ratio of 2. In fact, the percentages of complexed pDNA were more than 90% at N/P ratio of 2, and more than 95% at N/P ratio of more than 4. On the other hand, the percentages were more than 70% at N/P ratio of 0.5 and just under 90% at N/P ratio of 1. The changes in the size and the ZP of ternary complexes at the N/P ratio of more than 4 may be due to the degree of compaction. Stable polyplexes of RVG-R9 and pDNA without PEG-PEI were formed at N/P ratios of 20 or larger (N/P 10: 274 ± 7.0 nm, N/P 20: 110.1 ± 0.1 nm). The polyplexes without PEG-PEI were positively charged, and aggregation was observed with increasing pDNA. Moreover, stable polyplexes of PEG-PEI and pDNA without RVG-R9 could be formed at N/P ratios of 10 or larger, and the surface potentials of the polyplexes were almost neutral (N/P 10: 0.17 ± 0.55 mV, N/P 20: 0.19 ± 0.32 mV). These results suggest that the combination of RVG-R9 and PEG-PEI enables the formation of stable cationic ternary complexes with a small amount of cationic materials. These differences in the zeta potentials also suggested that the RVG-R9 peptide was exposed at the surface of the ternary complexes even though it was in the presence of PEG. In fact, the gene delivery effects on Neuro2a cells by ternary complexes at an N/P ratio of 10 were enhanced with increasing ratio of PEG-PEI, although the effects were not as high as the effects by LF2000. On the other hand, significant gene expression by ternary complexes at an N/P ratio of 10 was not observed in HeLa cells, although the effect by LF2000 was comparable with that in Neuro2a. This was thought to be due to both features of the ternary complexes, namely the stability by PEG, and the targeting ability by RVG. To confirm whether the interaction occurred through RVG-R9, polyplexes without RVG-R9 (N/P 10) were used, and the interaction of the polyplexes without RVG-R9 and Neuro2a cells was observed less. This was thought to be due to the absence of the targeting moiety to cells and the zeta potential of the polyplexes, which was almost neutral. Therefore, the polyplexes without RVG-R9 might not interact with neuronal cells, even if the polyplexes could be passed through the BBB by NBs and FUS. Furthermore, pretreatment with an excess amount of RVG peptide (about 20- or 40-fold) compared to the amount used in the transfection experiment significantly reduced gene expression in a dose-dependent manner, although the nACh receptor occupancy with peptide was unclear. These results suggested that the interaction and transfection specifically to neuronal cells could occur via the RVG peptide.

We previously mentioned that a transient BBB opening could be induced by the combination of NBs and FUS, resulting in the delivery of nucleic acids to the focused brain site [19]. In this study, we attempted to use another equipment to apply FUS at a lower intensity. The change in ultrasound equipment also changes the conditions, such as the focal length and acoustic output. Factors that affect the ultrasound energy include the target tissues, medium materials, such as water and gel, and temperature. Therefore, optimization is essential when the experimental conditions are changed. In this study, we evaluated the extravasation of EB, gene expression, and hemorrhage in the FUS-exposed site to determine the optimal conditions for transfection by the combination of ternary complexes, NBs, and FUS. As a result, gene expression increased in an intensity dependent manner. Furthermore, the tissue damage was strongly influenced by the duty cycle, which is the ratio between the pulse duration and the pulse interval, and could be suppressed by increasing the pulse repetition frequency. Based on a previous report, the duty cycle is highly correlated with tissue damage, and pulse repetition frequency influences BBB opening without conspicuous tissue damage [39]. These study results are partly consistent with the findings of our study. In this study, we showed that the luciferase gene expression level in the group treated with the combination of ternary complexes, NBs, and FUS was significantly higher than that of the control mice group injected buffer only or injected ternary complexes only. However, there was no significant difference between the right and left cerebral hemispheres in the FUS-treated mice. We are further investigating the adequate exposure method and FUS conditions for gene delivery to a limited area. In future, we need to perform a histochemical analysis of the brain as well as the other organs in detail, such as the evaluations of tissue damage and accumulation of the nanocarrier, and determine the safe and effective conditions of FUS and ternary complexes. Furthermore, it would be also valuable to evaluate the influences of size on the delivery effects and the safety using microbubbles that are commercially available and in a clinical pathway.

In the brains of mice injected with ternary complexes that were not exposed to FUS, gene expression was not observed. Under the conditions of this study, the gene expression region could be partly controlled by FUS exposure even when the RVG-modified ternary complexes were injected. Systemic injection of ligand-modified carriers might influence unwanted areas when the target of the ligand is expressed in various tissues. However, the combination of physical methods, such as ultrasound, could lead to an increase in the efficiency of delivery to target tissue and a decrease in the influence on other tissues. In previous studies, we developed pDNA, siRNA, or miRNA-loaded NBs using cationic lipids to increase the efficiency of systemic delivery [25,26,27,28]. Loading ternary complexes onto anionic NBs might result in further improvements in the efficiency of gene delivery. In the future, we will attempt to identify the cells transfected with pDNA in the brain tissue and confirm whether the efficiency of gene delivery to neuronal cells increased by the method described in this study. Gene delivery specific to target cells in the brain tissues could be a promising treatment for CNS diseases, such as Parkinson’s disease, Alzheimer’s disease, amyotrophic lateral sclerosis (ALS), and ischemic stroke. Our method could be useful for gene therapies to target malignant brain tumors by the combination of tumor specific targeting ligand. We would like to perform therapeutic gene transfection experiments, which could lead to the development of a safe and useful therapeutic system for CNS diseases.

## 5. Conclusions

This study showed that ternary complexes interacted specifically with Neuro2a cells via the RVG peptide. Furthermore, we demonstrated that the combination of ternary complexes, NBs, and FUS could deliver pDNA to the brain tissue only in the FUS-exposed area. These results suggest that gene delivery to the brain by the combination of the targeting carrier and ultrasound energy may be useful for the treatment of CNS diseases.

## Figures and Tables

**Table 1:** Size and zeta potential of ternary complexes.

N/P	Mean Size (nm)	PDI *	Zeta Potential (mV)
2	320.0 ± 55.4	0.203 ± 0.03	4.4 ± 3.0
4	106.3 ± 12.9	0.159 ± 0.01	13.2 ± 4.4
6	100.7 ± 6.0	0.181 ± 0.11	14.6 ± 3.8
8	101.5 ± 23.6	0.149 ± 0.07	12.0 ± 0.9
10	115.4 ± 19.4	0.253 ± 0.08	13.6 ± 6.1

* PDI: polydispersity index.

**Table 2:** Size and zeta potential of ternary complexes at the N/P ratio of 10.

PEG-PEI:RVG-R9	Mean Size (nm)	PDI	Zeta Potential (mV)
2:8	100.3 ± 3.5	0.277 ± 0.15	14.7 ± 9.0
5:5	115.4 ± 19.4	0.253 ± 0.08	13.6 ± 6.1
8:2	123.9 ± 32.2	0.188 ± 0.04	12.0 ± 2.7

## Data Availability

Not applicable.

## Supplementary Materials

The following are available online at https://www.mdpi.com/article/10.3390/pharmaceutics13071003/s1, Figure S1: The formation of ternary complexes composed of pDNA, PEG-PEI, and RVG-R9 peptide at N/P ratio of 10, Figure S2: Cell viability after the transfection with the ternary complexes.
