# Supplementary material for: Ternary Complexes of pDNA, Neuron-Binding Peptide, and PEGylated Polyethyleneimine for Brain Delivery with Nano-Bubbles and Ultrasound
Source: Pharmaceutics. 2021 Jul 1;13(7):1003. doi: 10.3390/pharmaceutics13071003 (PMC8309135; doi:10.3390/pharmaceutics13071003)
Supplement: Supplementary file 1 [file pharmaceutics-13-01003-s001.zip › pharmaceutics-1223383-supplementary.pdf]

pharmaceutics

Supplementary Materials: Ternary Complexes of pDNA, Neuron-Binding Peptide, and PEGylated Polyethyleneimine for Brain Delivery with Nano-Bubbles and Ultrasound

Yoko Endo-Takahashi, Ryo Kurokawa, Kanako Sato, Nao Takizawa, Fumihiko Katagiri, Nobuhito Hamano, Ryo Suzuki, Kazuo Maruyama, Motoyoshi Nomizu, Norio Takagi and Yoichi Negishi

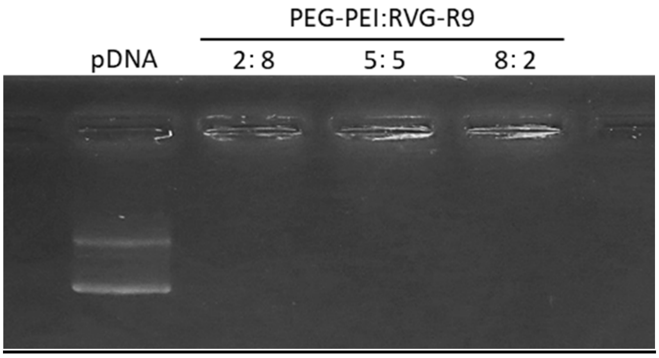

Figure S1. The formation of ternary complexes composed of pDNA, PEG-PEI, and RVG-R9 pepTable 10. A fixed amount of pDNA was incubated with various amounts of cationic polymer and peptide (PEG-PEI:RVG-R9 = 2:8, 5:5, and 8:2). The ternary complexes were analyzed using agarose gel electrophoresis.

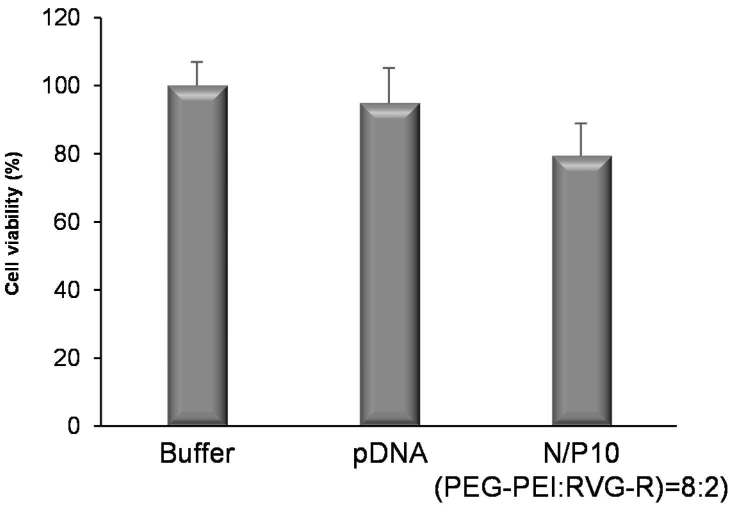

Figure S2. Cell viability after the transfection with the ternary complexes. Neuro 2a cells were incubated with the ternary complexes or pDNA (pDNA 1 μg/well) for 4 h. Cell viabilities were analyzed 2 days after the transfection. The bars show the mean and S.D. ($n = 3$).
